# Vibrational properties and bonding nature of Sb_2_Se_3_ and their implications for chalcogenide materials†
†Electronic supplementary information (ESI) available: Additional computational data and discussion. See DOI: 10.1039/c5sc00825e



**DOI:** 10.1039/c5sc00825e

**Published:** 2015-06-29

**Authors:** Volker L. Deringer, Ralf P. Stoffel, Matthias Wuttig, Richard Dronskowski

**Affiliations:** a Institute of Inorganic Chemistry , RWTH Aachen University , Landoltweg 1 , 52056 Aachen , Germany . Email: drons@HAL9000.ac.rwth-aachen.de ; Fax: +49 241 80 92642; b Institute of Physics IA , RWTH Aachen University , 52056 Aachen , Germany; c Jülich–Aachen Research Alliance (JARA-FIT and JARA-HPC) , RWTH Aachen University , 52056 Aachen , Germany

## Abstract

Antimony selenide (antimonselite, Sb_2_Se_3_) is a versatile functional material with emerging applications in solar cells. It also provides an intriguing prototype to study different modes of bonding in solid chalcogenides, all within one crystal structure. In this study, we unravel the complex bonding nature of crystalline Sb_2_Se_3_ by using an orbital-based descriptor (the crystal orbital Hamilton population, COHP) and by analysing phonon properties and interatomic force constants. We find particularly interesting behaviour for the *medium-range* Sb···Se contacts, which still contribute significant stabilisation but are much softer than the “traditional” covalent bonds. These results have implications for the assembly of Sb_2_Se_3_ nanostructures, and bond-projected force constants appear as a useful microscopic descriptor for investigating a larger number of chalcogenide functional materials in the future.

## Abstract 2

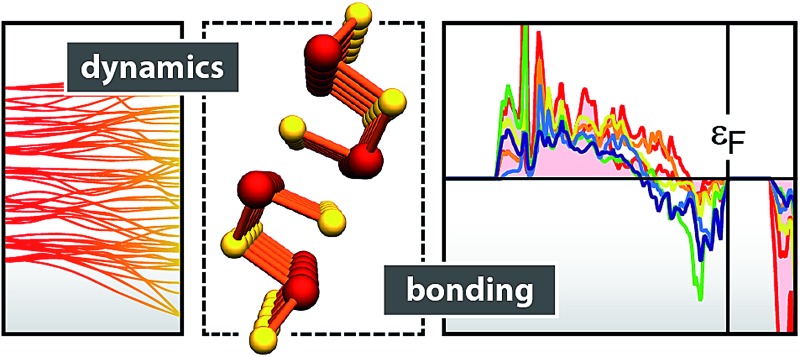
There is more to chemical bonding in chalcogenides than the shortest, strongest bonds, as revealed by microscopic quantum-chemical descriptors.

## Introduction

Chalcogenide materials continue to attract widespread attention, which is largely due to versatile technological applications.^1–6^ The antimony chalcogenides Sb_2_ *Ch*_3_ (where *Ch* denotes S, Se, or Te) are prime examples: Sb_2_S_3_ is used in organic–inorganic hybrid solar cells,^1^ and a computational study suggested that the heavier Sb_2_Se_3_ might allow for even higher conversion efficiency;^2^ subsequently, a multitude of prototype solar cells based on Sb_2_Se_3_ have been reported.^3^ The heavier Sb_2_Te_3_ is a key ingredient for phase-change data-storage materials^4^ and has more recently drawn massive interest due to its topologically insulating nature.^5^ Finally, antimony chalcogenides and their alloys have long been renowned as thermoelectric materials.^6^ Given such diverse applications, it is vital to closely understand the microscopic nature of these compounds to enable further developments.

Antimony selenide (Sb_2_Se_3_), first described in the 1950s,^7^ occurs naturally in the mineral antimonselite.^8^ The crystal structure^9^ is isomorphous to Sb_2_S_3_ and will be dissected in detail below. The electronic properties of Sb_2_Se_3_ have been thoroughly studied by means of density-functional theory (DFT), with emphasis on electron density,^10^ elastic properties and band gaps;^11^ the latter were subsequently computed by inclusion of *GW* corrections,^12^ which is crucial for the above-mentioned applications in photovoltaics.^2,3^ Electronic-structure trends within the homologous series from Sb_2_O_3_ to Sb_2_Te_3_ were discussed very recently, too.^13^ Regarding *vibrational properties* of Sb_2_Se_3_, on the other hand, there is a visible gap in the DFT literature between previous reports on Sb_2_S_3_ (ref. 14) and Sb_2_Te_3_,^15^ respectively. We started out aiming to fill this gap.

**Fig. 1:**
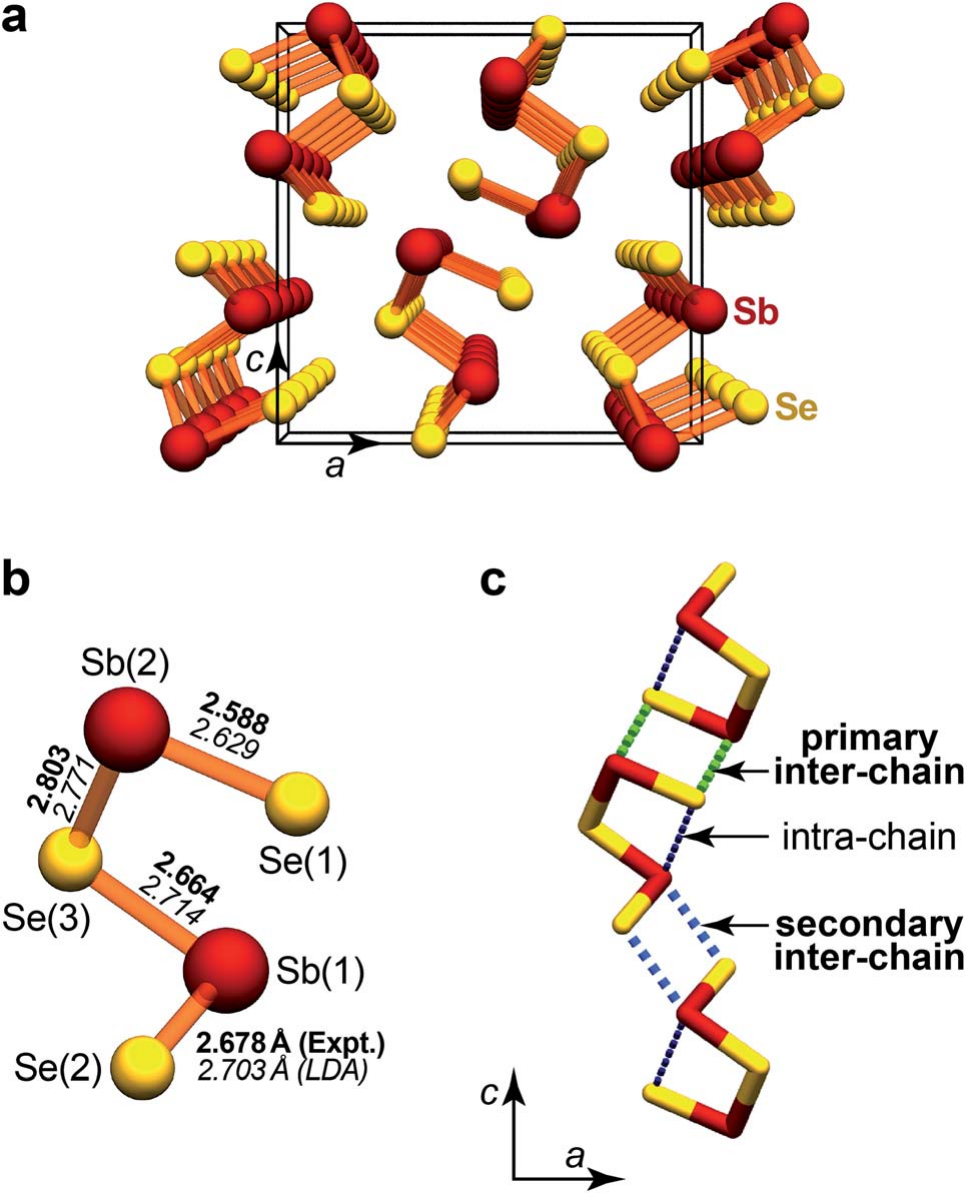
Crystal structure of Sb_2_Se_3_.^9*b*^ (a) Perspective view down the short *b*-axis, highlighting the “infinite chains” that extend through the crystal, with only the short bonds drawn. (b) Fragment from the above chain, with atomic labelling as in ref. 9*b*, and Sb–Se distances in Å from experiment (boldface; ref. 9*b*) and theory (italics; this work). (c) Structural drawing to emphasise the “weak” contacts along the *c*-axis, which connect the strongly bonded 1D chains.

From a crystal-chemical point of view, Sb_2_Se_3_ is likewise a most intriguing material. Its unit cell exhibits a very low-symmetric environment both of the antimony and the selenium atoms, and the structure is conventionally described in terms of one-dimensional “chains” along the direction of the *b*-axis.^9*a*^ Furthermore, there are atomic contacts *between* these fragments which link the 1D chains to give the 3D orthorhombic structure (Fig. 1). This bonding anisotropy is directly relevant for recent reports on nanoribbons and nanowires of Sb_2_Se_3_:^16^ the latter are cleaved from the bulk phase, in a way controlled by physical and chemical interactions, and such nanostructures are of great interest for applications—owing, for example, to improved photosensitivity.^16*e*^

Linking fundamental solid-state chemistry and applications in such a way, crystalline Sb_2_Se_3_ is an interesting model system which contains Sb–Se bonds of different length and (presumably) strength, all within one and the same unit cell. This idea of exploring a large number of different contacts in a single structure has been used before, albeit in a different context: namely, with regard to charge-density descriptors of different hydrogen bonds in solids^17^ and to their covalency.^18^ Here we show how such concepts can be transferred to chalcogenide chemistry, and what can be learned from them.

## Computational methods

DFT computations were performed using the projector augmented-wave (PAW) method^19^ as implemented in the Vienna *ab initio* Simulation Package (VASP).^20^ Unless mentioned otherwise, exchange and correlation were modelled in the local density approximation (LDA),^21^ which recently proved an economic choice for the lattice dynamics of the heavier homologue Sb_2_Te_3_ (ref. 22) and was validated there against earlier nuclear inelastic scattering (NIS) experiments. In particular, it was shown in ref. 22 that the force constants measured by NIS can be reproduced with high accuracy by the simple LDA. Nonetheless, we have here performed additional computations with higher-rung DFT methods for further validation: different functionals in the generalised gradient approximation (GGA),^23^ a number of methods to account for dispersion interactions,^24^ and, finally, *meta*-GGA computations;^25^ details of all these computations are provided in the ESI.†

In all these computations, the cutoff energy for the plane-wave expansion was 300 eV, and reciprocal-space integration was performed on dense Monkhorst–Pack meshes (sized 4 × 12 × 4 for bulk cells, and 1 × 12 × 1 for supercells of 1D structures).^26^ Stringent convergence criteria were set, to minimise energy differences below 10^–8^ (10^–6^) eV per cell between electronic (structural) cycles, respectively, and an additional support grid for augmentation charges was activated to improve numerical precision.

Phonon computations were done using the Parlinski–Li–Kawazoe method^27^ as implemented in PHONOPY,^28^ with supercells corresponding to 2 × 6 × 2 expansions of the unit cell (thus exceeding the requirements found in ref. 14 for Sb_2_S_3_). The interatomic forces were obtained from VASP using the *Γ*-point approximation, and the methodology largely follows our previous studies on the lattice dynamics of GeSe^29^ and Sb_2_Te_3_.^22^ The reciprocal-space mesh for the evaluation of the vibrational eigenvalues was 8 × 24 × 8, which led to well-converged phonon densities of states.

To analyse computed electronic structures in a chemical language, the concept of bonding between atoms is a most valuable one.^30^ In the solid state, a number of tools have been conceived for this purpose, which partition computed properties such as the electronic density into “bonding” (stabilising) and “antibonding” (destabilising) interactions between neighbouring atoms. In the 1980s already, Hughbanks and Hoffmann introduced the iconic crystal orbital overlap population (COOP) method,^31^ using the overlap of adjacent valence orbitals, *S*_*μν*_ = *μ*|*ν* to gauge the nature and strength of chemical interactions. For periodic DFT computations, the crystal orbital Hamilton population (COHP) method has been subsequently proposed, which is based on a partitioning of the (one-particle) band-structure energy, and in this case the criterion for bonding is given by the expectation value of the Hamiltonian, *H*_*μν*_ = *μ*|*H*|*ν*.^32^ An interesting discussion of partitioning schemes for analysing bonding has been given by Glassey and Hoffmann.^33^ COHPs have been previously used with success to study bonding in binary^22,34^ and ternary chalcogenide materials,^35^ and they are also the method of choice for the present work. Technically, COHPs were obtained from tight-binding linear muffin-tin orbital (LMTO) theory, in which the above-mentioned quantities are directly accessible in terms of orbital combination coefficients. LMTO computations were performed using the LDA functional of von Barth and Hedin^36^ and the atomic spheres approximation (TB-LMTO-ASA program).^37^

## Results and discussion

### Atomic and electronic structure

The structure of Sb_2_Se_3_ has been studied by single-crystal X-ray diffraction before.^9^ Our computations reproduce the lattice parameters reasonably well (Table 1), given underestimation as is typical for LDA-DFT; the results are in line with a previous report at a comparable level of theory.^12*b*^ The overall quality of the structural description may be assessed by the directionally resolved root mean-square displacement (rms; the lower, the better),^38^ which amounts to rms_*x*_ = 0.07 and rms_*z*_ = 0.03, respectively; rms_*y*_ equals zero due to special sites.

**Table 1:** Lattice parameters of Sb_2_Se_3_ (space group *Pnma*, no. 62)

	Expt. (XRD; ref. 9*a*)	Expt. (XRD; ref. 9*b*)	DFT-LDA (This work)
*a* (Å)	11.77(1)	11.7938(9)	11.534
*b* (Å)	3.962(7)	3.9858(6)	3.960
*c* (Å)	11.62(1)	11.6478(7)	11.221

An interesting detail lies in the computed Sb–Se bond lengths, of which we visualise the shortest ones in Fig. 1b: the Sb(1)–Se(3) bond in XRD is slightly shorter than its counterpart Sb(1)–Se(2) (Δ*d* = –0.014 Å), while DFT reverses this order (Δ*d* = +0.011 Å). As such, this is not worrisome since the differences are small, and the computation refers to “zero Kelvin” whereas both experiments have been conducted at ambient temperature. The difference in experimental and computed distances is larger for the longer Sb–Se contacts that we will discuss below. At this point already, we remark that a low-temperature diffraction experiment could easily clarify the issue.

**Fig. 2:**
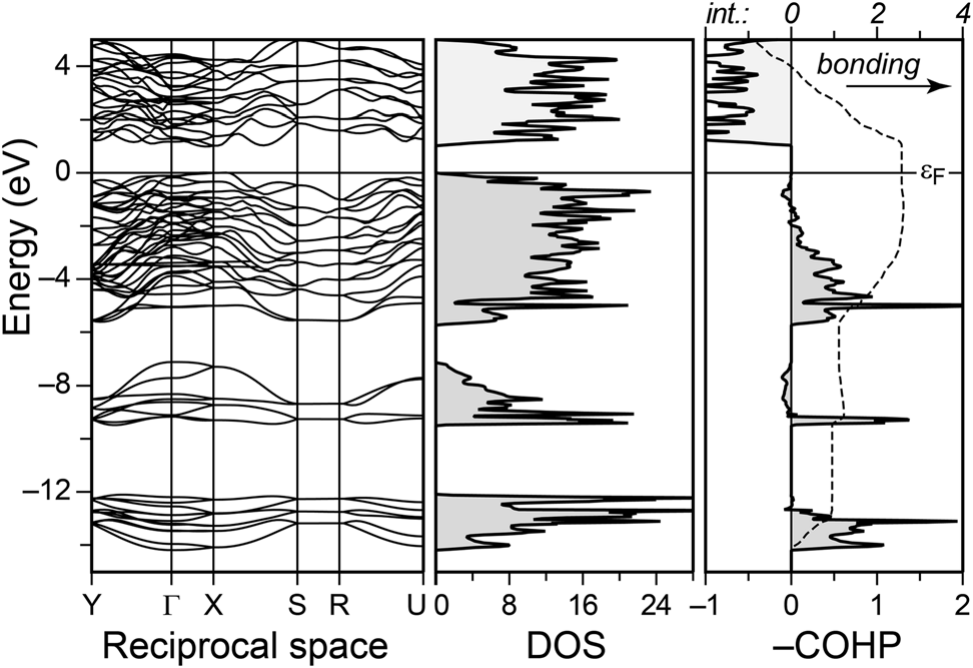
TB-LMTO-ASA electronic band structure and densities of states (DOS) for Sb_2_Se_3_, and exemplary crystal orbital Hamilton population (COHP) curve for the short Sb(2)–Se(1) contact. The energy integral (“int.”) is given by a dashed line. Negative expectation values of the Hamiltonian reflect stabilisation, and thus we plot –COHP as is convention.^30*c*^ The Fermi level *ε*_F_ is set as the energy zero.

The electronic structure of Sb_2_Se_3_ is that of a typical semiconductor and has been studied at a high level of theory (DFT + *GW*).^12^ We do not aim to reproduce these costly computations here; for reference, however, we show the LDA bands and densities-of-states (DOS) in Fig. 2. The present work, instead, is concerned with the *bonding nature*, and the principle is illustrated in the same figure. By singling out pairwise contributions to the band-structure energy, we perform COHP analysis,^30c,32^ in which bonding (stabilising) contributions are visualised on one side of the energy axis, and antibonding (destabilising) ones on the other.

The COHP for the shortest bond, Sb(2)–Se(1), is shown in Fig. 2b (right): unambiguously bonding interactions dominate the entire range of valence bands, with small antibonding regions around –8 and –0.5 eV, respectively. The importance of the latter is minor, however, as easily seen in the energy-integrated populations (dashed line): starting at the bottom of the valence bands, the integral rises almost continuously and reaches a stabilising value of >2.5 eV at the Fermi level. The latter integral serves as an indicator toward the covalent bond strength, albeit both are not directly superimposable.^30c,32^

### Bonding nature from COHP analysis

As said above, there is more to the structural chemistry of Sb_2_Se_3_ than the closest contacts which formally make up the 1D chains. In particular, we will here address the role of the *medium-length* Sb–Se contacts, which link the chains along the crystallographic *c* axis. There are three distinguishable inter-chain contacts of this type, as drawn in Fig. 1c; according to the experimental distances, we label them as “primary” (3.007 Å) and “secondary” (3.247 Å), respectively. There is also an additional Sb(1)–Se(1) contact in each of the chains (dubbed “intra-chain”; *d*_Sb–Se_ = 3.215 Å).

We summarise the ICOHP values in Table 2. The above-mentioned differences in structural descriptions between experiment and theory become apparent there, too: while the variation in the short Sb–Se distances is marginal, the *medium-range* contacts are significantly compressed in the LDA-DFT optimised structure, and the *inter-chain* contact Sb(1)–Se(2)’ is now slightly shorter (3.049 Å) than its counterpart Sb(1)–Se(1) within the chains (3.077 Å). Interestingly, the latter attains the less stabilising –ICOHP value, no matter if the experimental or optimised structure serves as input for the single-point LMTO computation.^39^ Hence, there is unambiguously stronger interaction *between* the chains in the medium-range regime.

**Table 2:** Bond lengths and corresponding integrated COHP values (–ICOHP) for all relevant Sb–Se contacts in crystalline Sb_2_Se_3_ ^*a*^

	Experimental structure^9b^		Optimised structure^*a*^	
	*d*_Sb–Se_ (Å)	–ICOHP (eV)	*d*_Sb–Se_ (Å)	–ICOHP (eV)
Sb(2)–Se(1)	2.588	2.84	2.629	2.57
Sb(1)–Se(3)	**2.664**	2.21	**2.714**	1.89
Sb(1)–Se(2)	**2.678**	2.19	**2.703**	2.00
Sb(2)–Se(3)	2.803	1.70	2.771	1.73
Sb(2)–Se(1)^*b*^	3.007	0.74	2.977	0.83
Sb(1)–Se(1)^*c*^	**3.215**	0.24	**3.077**	0.51
Sb(1)–Se(2)^*b*^	**3.247**	0.37	**3.049**	0.66
Sb(2)–Se(2)^*d*^	3.486	0.10	3.355	0.15
Sb(1)–Se(3)^*d*^	3.739	0.00	3.495	0.07

^*a*^Bonds whose sequence is inverted during optimisation have been highlighted in boldface.

^*b*^Between chains, in direction of the *c*-axis (*cf.* Fig. 3).

^*c*^Longer contact within one chain.

^*d*^Between chains, in direction of the *a*-axis.

**Fig. 3:**
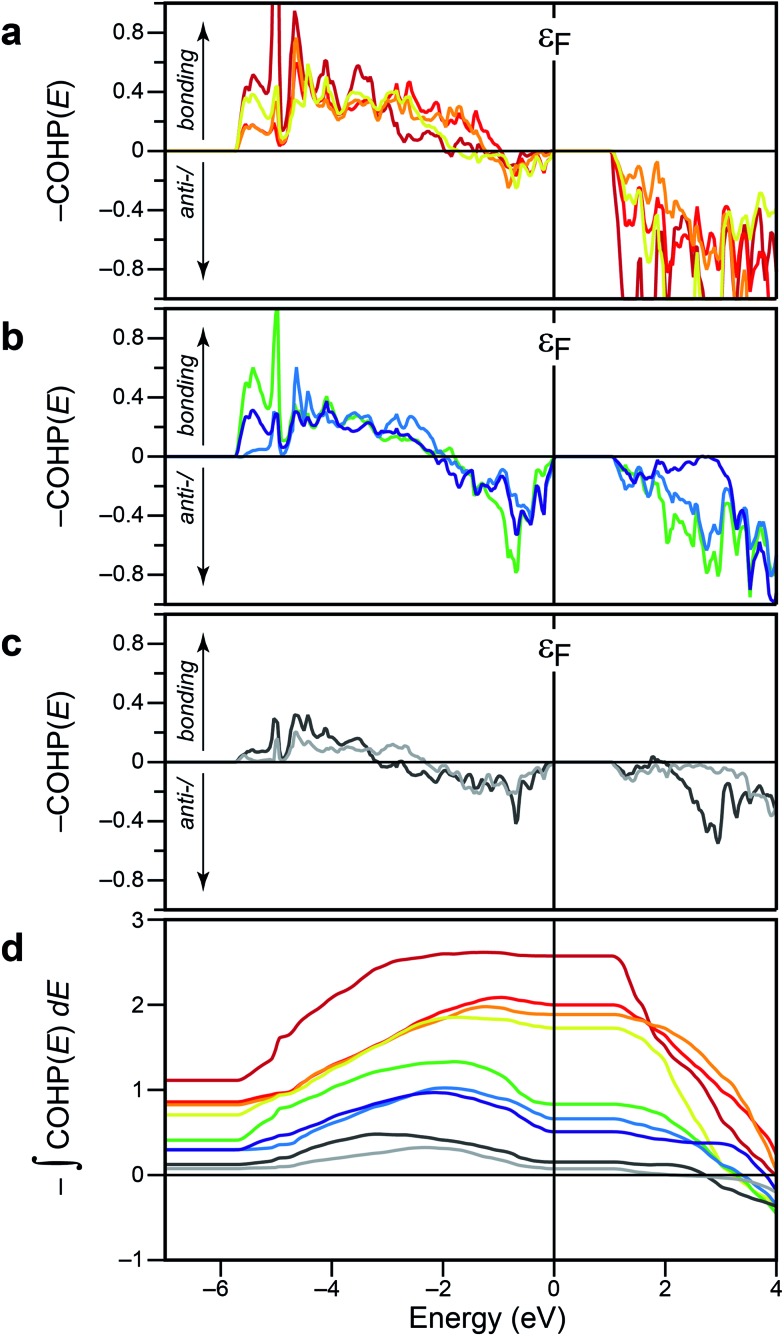
(a–c) COHP curves for all relevant Sb–Se contacts in the optimised structure, grouped, from top to bottom, according to strong, weaker, and nonbonding contacts (see text). (d) Energy integrals of the above –COHP(*E*) data; colours refer to individual bonds as in the panels above. At variance with Fig. 2, we here set the energy axis as the *horizontal* one, for easier interpretation of the integral values.

Fig. 3 collects the energy-resolved COHP curves for all these Sb–Se contacts, which affords a more detailed look into the electronic structure. The bonding “fingerprints” found in crystalline Sb_2_Se_3_ can be classified into three groups. First, there are three short, clearly covalent bonds in the 1D chains (*d* ≤ 2.9 Å), and they exhibit almost no antibonding contributions up to *ε*_F_ (Fig. 3a), not surprisingly.

Second, there is a group of the aforementioned “weak” contacts along the *c*-axis (*d* ≤ 3.3 Å). These, by contrast, show significant antibonding contributions at the top of the valence band, that is, from –2 eV to the Fermi edge (Fig. 3b). The integrals as plotted below get lowered while crossing this area, but their total amounts at *ε*_F_ still indicate significant stabilisation in the 50–80 kJ mol^–1^ range. We note that occupied, antibonding levels have been identified in crystalline and amorphous GeTe and in related phase-change data-storage materials,^34,35,40^ and also in Sb_2_Te_3_.^22^ A direct comparison, however, would be premature at this point.

Finally, there are the longer contacts (*d* ≤ 3.8 Å), for which COHP curves are shown in Fig. 3c. These exhibit what was previously suggested as a fingerprint of noncovalent interactions:^22^ there is some stabilisation, up to ≈–3 eV, but this region is then counteracted by an approximately equally large antibonding area. This is also reflected in the integrals which almost drop back to zero (Fig. 3d). The overall magnitude of the COHP curves is significantly smaller, mirroring the diminishing degree of orbital overlap at larger interatomic distances.

**Fig. 4:**
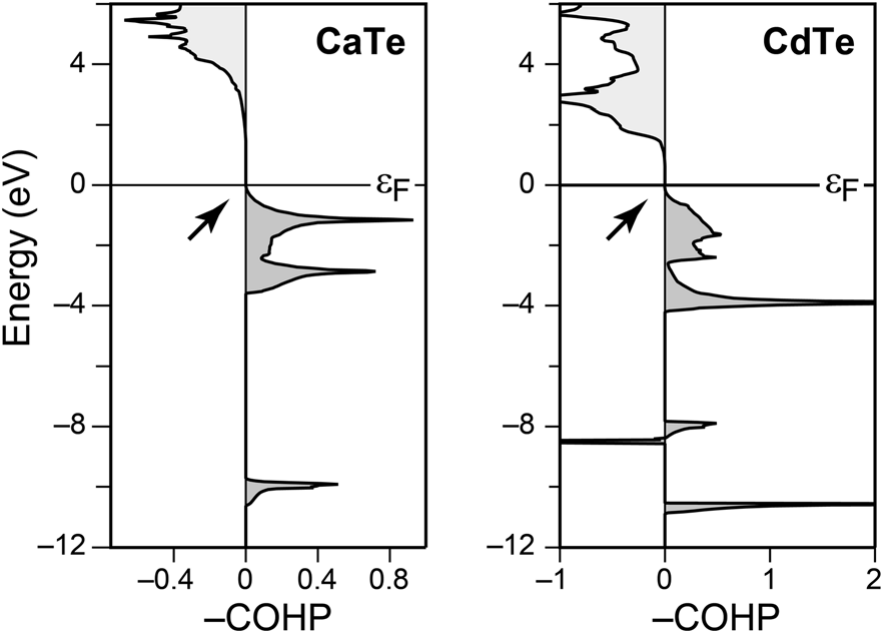
COHP analysis for the nearest-neighbour contacts in two prototypical telluride materials, *viz.* rocksalt-type CaTe (left) and zincblende-type CdTe (right); structural data from ref. 42 and 43, respectively. The antibonding peak slightly below –8 eV in the CdTe case stems from Cd 4d–Te 5p interactions, as revealed by an orbital-resolved analysis (omitted for brevity). The top of the valence band, however, is devoid of antibonding interactions in both cases (see arrows).

Concluding the present section, we stress that the most diverse behaviour seen in Fig. 3 all stems from heteropolar Sb–Se bonds within *one single* crystal structure. This is at variance with simple III–V semiconductors (take GaAs), which are derived from diamond by iso-valence-electronic substitution and show fully optimised covalent bonding; COHPs for both materials are given, *e.g.*, in ref. 41. Likewise, neither CdTe nor rocksalt-type calcium telluride (CaTe) exhibit antibonding COHPs at the top of the valence band, as we show in Fig. 4.

### Lattice dynamics

**Fig. 5:**
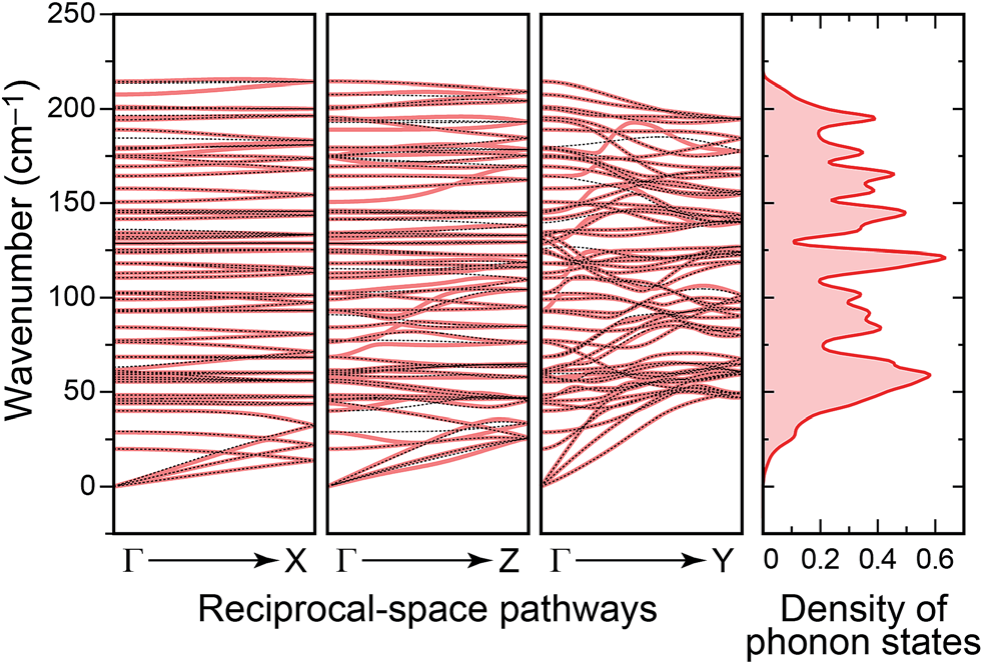
Phonon dispersions computed for crystalline Sb_2_Se_3_ along representative high-symmetry lines. The increasing degree of dispersion upon going from the “weakly” to “strongly” bonded directions is clearly visible and reflects the anisotropy of the underlying crystal structure. Results with non-analytical term correction^41^ (thin dotted lines) and without this correction (pale red) are largely superimposable along *Γ* → X and *Γ* → *Z*, less so along *Γ* → *Y*. On the right-hand side, the computed density of phonon states (DPS) is seen.

We now move on to study the vibrational properties of crystalline Sb_2_Se_3_. Computed phonon dispersions along important high-symmetry directions in reciprocal space, and also the total density of phonon states (DPS) are shown in Fig. 5. The crystal lattice is dynamically stable with no imaginary eigenvalues. There is no gap between sets of bands as is present, *e.g.*, in the somewhat related *Pnma* structure of GeSe.^29^

The vibrations range up to ≈200 cm^–1^, which lies between values computed for the lighter Sb_2_S_3_ (up to ≈320 cm^–1^)^14^ and the heavier Sb_2_Te_3_ (≈170 cm^–1^).^15^ The material is hence significantly softer than Sb_2_S_3_, as seen before in a comparative IR absorption experiment for the compound and its chemical relatives.^44^ There are also recent, most interesting high-pressure Raman scattering studies on Sb_2_Se_3_;^45^ the pressure domain, however, is not the topic of the present work. For comparison with previous and possible future experiments, atom-resolved partial DPS plots are discussed in the ESI.†

**Fig. 6:**
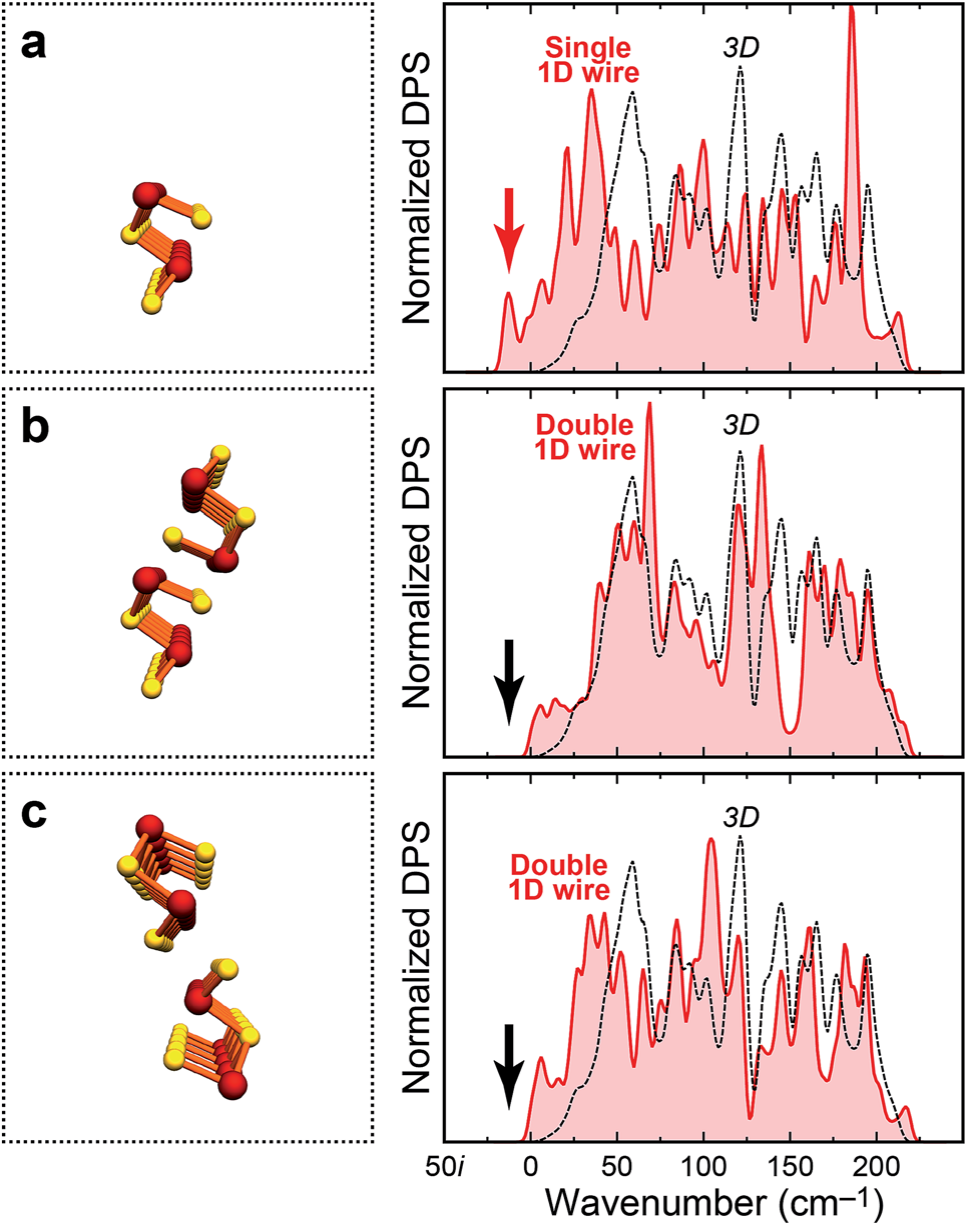
As Fig. 5, but for single and double 1D “wires” (the supercells employed are much larger than sketched here). The occurrence of imaginary phonon modes in the single wire (a) is emphasised by a red arrow. The double wires in panels (b and c), by stark contrast, are dynamically stable (black arrows). The spillover to marginally “negative” wavenumbers is an artifact of the broadening scheme.

Given the importance of *nanoscale* Sb_2_Se_3_, it seems useful not only to investigate the bulk material, but likewise lower-dimensional fragments derived from it. We have done so, *e.g.*, during methodologically related (supercell-based) studies of dimensionality in chalcogen-bonded crystals,^38^ and in earlier work on hydrogen-bond cooperativity.^46^ Similarly, we here start by computationally cleaving a single 1D wire (Fig. 6a) from the previously optimised crystal structure of Sb_2_Se_3_.^47^

The DPS for this isolated wire does exhibit imaginary modes (red arrow in Fig. 6a); it is hence *no* local minimum on the potential-energy surface. This is not unexpected since the structure has, on purpose, been quite “naively” cut from the crystal; it does not experience the environment in which it is usually found. Surprisingly, however, a double wire (Fig. 6b) exhibits *no* such problem and is dynamically stable: the difference between both is the additional presence of “weak” inter-chain contacts (*cf.* Fig. 1c). Similar dynamic stabilisation holds for the alternative double-wire structure containing the weaker, secondary inter-chain bonds (Fig. 6c). These model computations clearly underline the importance of the weak Sb–Se contacts, which is in line with the key message from COHP analysis (Fig. 3): there is more to Sb_2_Se_3_ than the “strong” covalent bonds.

We stress that our computations thus far refer to ideal structures *in vacuo*, whereas in experiment, surface reconstructions, reactivity, and possible ligands play important roles.^48^ Future work on this by combination of theory and experiment would seem highly rewarding.

### A link between covalency and forces

So far, this study has been concerned with two themes—first, with the covalent bonding as assessed by an orbital-based indicator; second, with the lattice dynamics that are grounded on interatomic forces. It seems interesting, finally, to link these two topics.

The most basic information that underlies the phonon computations is the forces and *force constants* for the different atoms. While average force constants can be obtained from experiment, theorists are in the advantageous position of having force-constant *matrices* available, and this allows one to spatially resolve particular interactions. Indeed, the study of bond force constants is an established concept in the chemistry of *molecules*,^49^ and has allowed for recent interesting applications: for example, identifying kinetically labile bonds^50^ or quantifying extremely strong ones.^51^ Also, interatomic force-constants have been quantified recently in a study of long-range interactions in thermoelectric materials.^52^ To investigate force constants in crystalline Sb_2_Se_3_, we here project the DFT-derived force-constant matrix Φ_*ij*_ on the unit vector along each bonding direction *d*_*ij*_, to arrive at a quantity best comparable to the notion of a bond force constant; the result is henceforth denoted “bond-projected” force constant *φ*_B_:1
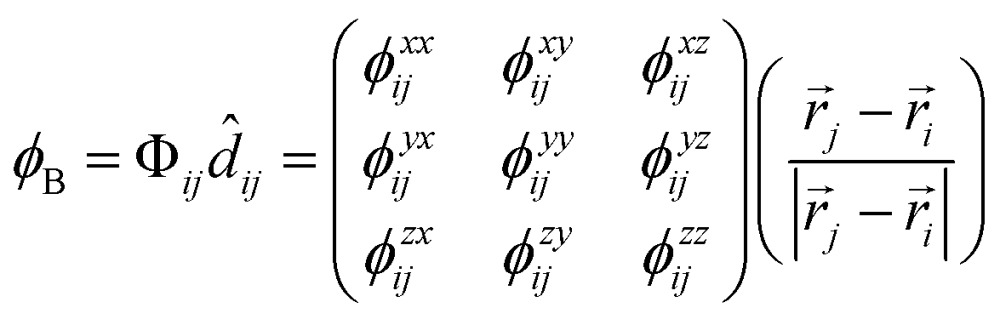
where the force constant is defined such that the *i*-th atom (here, Sb) is displaced and exerts force on the *j*-th atom (here, Se). Hence, *φ*_B_ is obtained for each bond individually, and may be compared to the respective COHP integral at *ε*_F_.

Before discussing these *ab initio* bonding descriptors, we round out the set of indicators by a very classical (and empirical) measure, which was introduced by Pauling in the 1940s.^53^ Therein, the bond length is expressed relative to the single A–B bond length (dubbed *D*_1_), by way of the bond number *n*:

2 *D*_*n*_ = *D*_1_ – 0.600 Å × log_10_ *n*

Tideswell *et al.* have applied precisely this tool to Sb_2_Se_3_ in their 1957 report on this compound.^9*a*^ How does it compare to the *ab initio* bonding descriptors used so far? We thus recall the sum of tabulated covalent radii (2.58 Å; ref. 53*a*), as Tideswell *et al.* have done, and inspect Pauling's bond number *n*, as in3
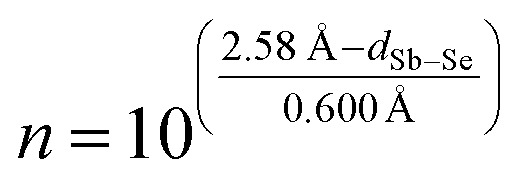



**Fig. 7:**
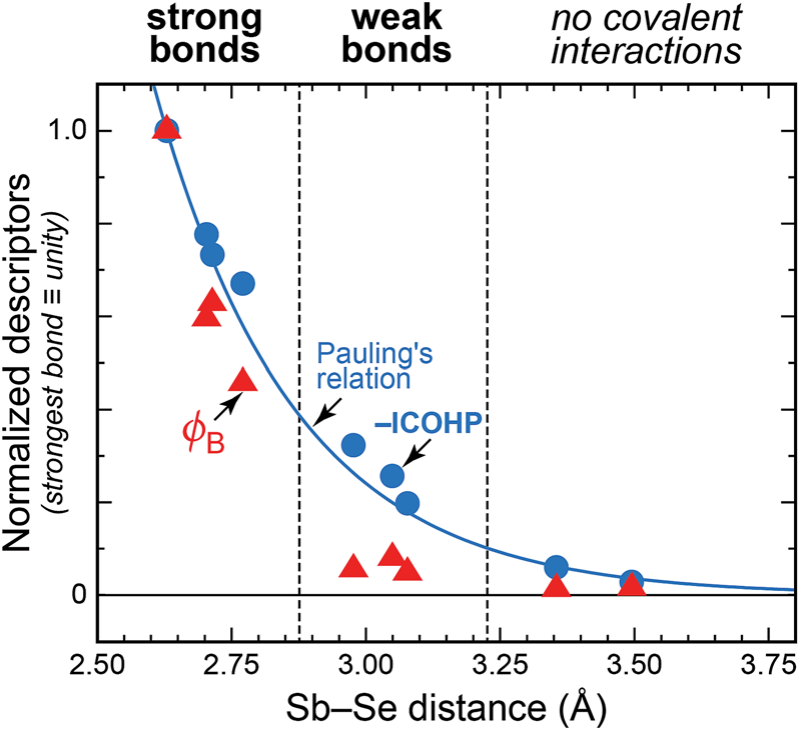
Bonding descriptors for Sb–Se contacts in crystalline Sb_2_Se_3_. Circles show integrated COHP values at *ε*_F_; triangles give values of bond-projected force constants (eqn (1)). For comparison, Pauling's measure of bond order (eqn (3)) is shown. All data are given on a normalised scale.

This finally leads to what is shown in Fig. 7: a plot comparing bond “stiffness” (*φ*_B_) and covalency (–ICOHP) for all relevant Sb–Se interactions in the solid structure. Thereby, all three descriptors have been normalised such that the shortest bond, Sb(2)–Se(1), obtains a value of 1.0. This plot may serve as an icon to summarise the different bonding modes in solid chalcogenides.

The integrated COHP values (blue circles in Fig. 7) correlate well with Pauling's empirical formula—up to a gratifying degree, in fact. The largest deviations on the normalised scale amount to 9% for the Sb(2)–Se(3) bond within the chains, and to ≈6% for both medium-range inter-chain contacts; in all other cases, the ICOHP data (circles) and Pauling's descriptor (blue line) practically coincide. The decay of the force constants (red triangles in Fig. 7), on the other hand, appears to be more rapid. The data points clearly fall into three groups. For the strong bonds shorter than ≈2.8 Å, both covalency and stiffness follow a similar trend, and for the longer contacts beyond ≈3.2 Å neither descriptor gives indication of bonding. In the intermediate regime, however, there remains significant covalency whereas the force constants drop rapidly.

It is crucial to ensure that this effect is not an artifact of a particular computational method. We therefore repeated the force-constant computations at several levels of theory and could unequivocally confirm the trend observed. For clarity, we limit our presentation to LDA results here, but provide data at the GGA, GGA-D, and *meta*-GGA levels as part of the ESI.†

The alert reader will interject that bond stiffness is not, conventionally, linked to the bond order, such that the presentation in Fig. 7 would be of limited value. However, we have likewise applied Badger's classical rule for bond length–force constant correlations:^49*a*^ the strong bonds obey it, but the weak bonds do not (see ESI†).

### Implications for chalcogenide materials

There are two important questions to make this study worthwhile beyond the particular case of Sb_2_Se_3_. First, can we transfer the effects observed to other compounds? Second, are the results relevant for other classes of functional materials?

**Fig. 8:**
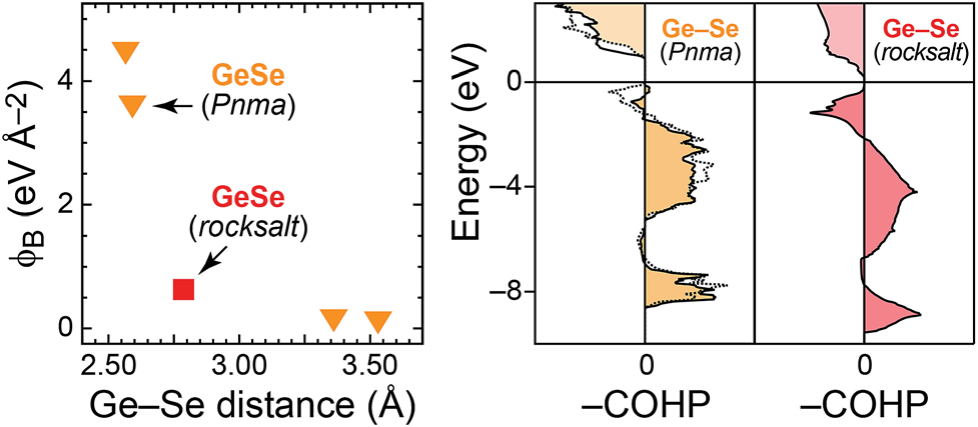
Bond-projected force constants and COHP analysis for the two GeSe polymorphs (structures and phonon computations for the latter taken from ref. 29; *φ*_B_ and COHP analysis: this work). There are two distinguishable, short Ge–Se contacts in the *Pnma* structure, for which COHPs are drawn with different line styles.

As regards the first question, the chemically related material GeSe seems particularly interesting. It takes two polymorphs: a layered structure, likewise in space group *Pnma*, and a rocksalt-type polymorph at elevated temperature. We have studied phonons in GeSe before^29^ and here extract *φ*_B_ from that dataset. The results are in Fig. 8, alongside COHP curves for both polymorphs. While the ground-state *Pnma* structure seems very similar to Sb_2_Se_3_, the rocksalt-type polymorph shows both characteristics assigned here to “weak” bonds: there are antibonding COHPs at the valence-band top, and the computed *φ*_B_ are significantly lower. Fig. 8 thus evidences that the method allows us to differentiate between polymorphs—an important property of *ab initio* bonding descriptors that we have asked for before.^54^

Let us finally answer the second question and look at another class of functional materials. The behaviour observed here—and the peculiarity in the weaker bonds—is in qualitative agreement with a previous model one of us proposed for the bonding nature of phase-change materials (PCMs) used in data storage.^55^ Therein, the amorphous, “classically” covalently bonded phase shows a rather steep potential (thus large bond force constants) whereas the crystalline phase exhibits a more shallow energy well.^55^ Both modes of bonding can, apparently, be reconciled with the COHP data in Fig. 3: the short bonds (Fig. 3a) are quite “classical” in their behaviour, whereas the longer ones exhibit antibonding admixtures (Fig. 3b), as do crystalline PCMs. It would now be interesting to apply the *φ*_B_ descriptor to a large number of candidate compounds, aiming, ultimately, to find new PCMs by “materials mapping”. A scheme developed recently for this purpose is in active use already at the present day.^56^

## Conclusions

A theoretical study of crystalline Sb_2_Se_3_ has afforded new insight into the chemical-bonding nature and vibrational properties of this important material. Phonons have been analysed for 3D and 1D networks of Sb_2_Se_3_: both contribute to the long-term goal of exploring the physical nature and chemical behaviour of the nanoscale material. The course of interactions—from strong to nonbonding—has been rationalised through COHP analysis and by inspection of bond-projected force constants. The latter seem to be an interesting descriptor for exploring a larger number of chalcogenide functional materials in the future.
