# Supplementary material for: Vibrational properties and bonding nature of Sb2Se3 and their implications for chalcogenide materials
Source: Chem Sci. 2015 Jun 29;6(9):5255–62. doi: 10.1039/c5sc00825e (PMC5669248; doi:10.1039/c5sc00825e)
Supplement: Supplementary file 1 [file SC-006-C5SC00825E-s001.pdf]

Electronic Supplementary Material (ESI) for Chemical Science.
This journal is © The Royal Society of Chemistry 2015

Electronic Supplementary Information (ESI) for

Vibrational properties and bonding nature of Sb_2Se_3 and their implications for chalcogenide materials

Volker L. Deringer,[a] Ralf P. Stoffel,[a] Matthias Wuttig[b,c] and Richard Dronskowski[a,c,]*

[a]Institute of Inorganic Chemistry, RWTH Aachen University, Landoltweg 1, 52056 Aachen, Germany

[b]Institute of Physics IA, RWTH Aachen University, 52074 Aachen, Germany

[c]Jülich–Aachen Research Alliance (JARA-FIT and JARA-HPC), RWTH Aachen University, 52056 Aachen, Germany

* Corresponding author. E-mail: drons@HAL9000.ac.rwth-aachen.de

Contents

Supplementary methods S2

DFT methods beyond the LDA S3

Supplementary results for bulk Sb_2Se_3 .... S7

Supplementary results for 1D chains S11

Structural raw data S13

Supplementary references S18

Supplementary methods

The data reported in the main text have been obtained in the local density approximation (LDA), not only due to its conceptual simplicity but also because the LDA has previously shown excellent performance in reproducing experimentally determined on-site force constants in the chemically related Sb_2Te_3.[S1] Nonetheless, the results reported in the paper must be independent of one particular DFT method and be reproducible at other, higher levels of theory. **We show in this ESI document that this is indeed the case.**

For this purpose, supplementary computations were performed using the VASP package as described in the main text, with the following methods (and otherwise comparable computational parameters):

- The generalised gradient approximation (GGA)—that is, the second rung of Perdew's "ladder" of DFT functionals[S2]—using, in particular,
    - the Perdew–Burke–Ernzerhof (**PBE**) functional;[S3]
    - the PBE functional revised for solids (**PBEsol**);[S4]
    - the Armiento–Mattsson (**AM05**) functional.[S5]

- Dispersion corrections to GGA, which have been seen to be important for layered tellurides and might also improve the description of Sb_2Se_3 (see also the discussion in Ref. S6):
    - the "D3" scheme of Grimme and co-workers,[S7] which is a pairwise *a posteriori* correction added to energies (and forces), both in the initially proposed zero-damping scheme (**D3** in the following) and
    - using Becke–Johnson damping (**BJ** in the following);[S8]
    - furthermore, the vdW-DF2 method of Langreth, Lundqvist, and co-workers (abbreviated as "**DF2**" in the following).[S9]

- Finally, the meta-GGA functional after Tao, Perdew, Staroverov, and Scuseria (**TPSS**) was employed to explore the effect of a "third-rung" functional (that is, one exceeding LDA and GGA in formal and methodological scope).[S10]

DFT methods beyond the LDA

Optimised lattice parameters. The structure of Sb_2Se_3 contains a range of "weak" interatomic contacts whose description is notoriously nontrivial for traditional DFT methods.[S11] We thus start by assessing the computed lattice parameters at different levels of theory; the results (for cells fully optimised at the respective computational level) are provided in **Figure S1** below. Not surprisingly, the cell volume in particular is underestimated by LDA, and overestimated by the PBE functional; some of the methods can alleviate this, but there is no clear "failure" of the LDA among its competitors. It is also interesting to note that along the *b*-axis, the deviations are mainly very small—this is the direction in which the covalently bonded chains extend. Along the *a*-axis, where only weak stacking interactions occur, the deviations are generally more pronounced. Finally, we stress that the aim of these data is *not* to benchmark DFT methods or judge their merit (note, *e.g.*, that several other formulations of "vdW-DF"-type functionals exist,[S9] some of which will likely provide better lattice parameters).

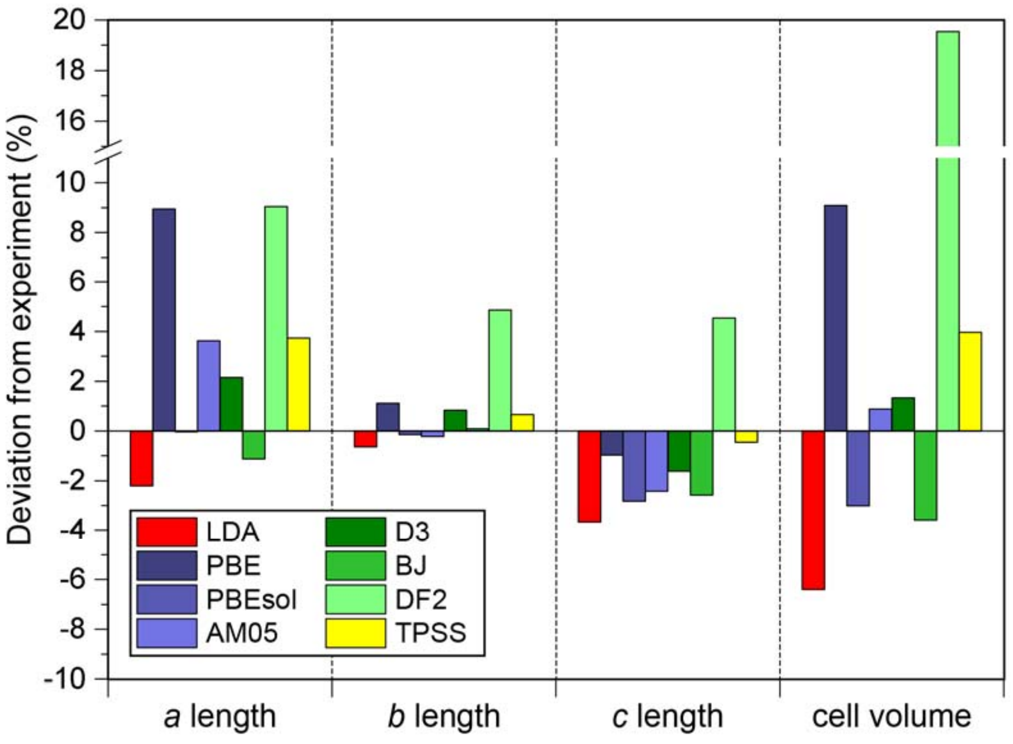

Figure S1. Quality of structural descriptions as judged from the computed lattice parameters and their deviation from experiment (single-crystal X-ray diffraction; data from Ref. S12).

Atomic positions. In addition to the computed lattice parameters, it is worthwhile to quantify the description of the individual atoms' positions; this gives more detailed insight into how well interatomic distances are reproduced at the different levels of theory. For this purpose, it has been suggested to inspect the root mean square (rms) deviation of experimental and computed Cartesian coordinates;[S13] later, this definition has been extended by George *et al.*[S14] and decomposed according to the different spatial directions, which seems particularly useful for anisotropic structures. By definition, the y component (rms_y) equals zero because all atoms reside on Wyckoff sites with $y = ¼$ or $y = ¾$. The results for the other two spatial directions (which are most important as the "weak" contacts occur in these) are provided in **Figure S2**. Again, certain improvements are possible, but there is not a clear failure of LDA, and we note that a minor deviation remains even with the best methods.

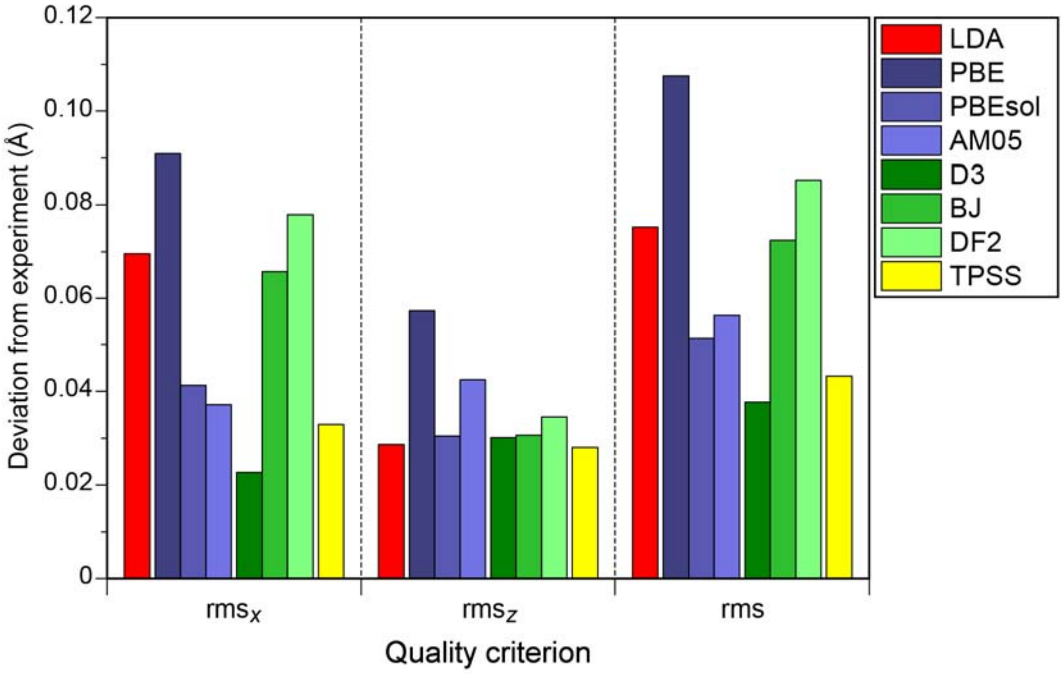

Figure S2. Quality of structural descriptions as measured by the directionally resolved and overall root mean square deviation of Cartesian coordinates.[S14]

Bond-projected force constants with different DFT methods. Among the key results of this study is a significant contrast in bond-projected force constants: they are large for the "classically" covalent bonds, but very low for the medium-range contacts. One might argue that more sophisticated formulations are required to capture properties of the latter, too, and hence we have repeated the phonon computations described in the main text at all levels of theory. The results—separated, for clarity, into two panels—are given below (**Figures S3** and **S4**), and they unambigously show that the conclusions obtained in the LDA hold also at the other DFT levels investigated.

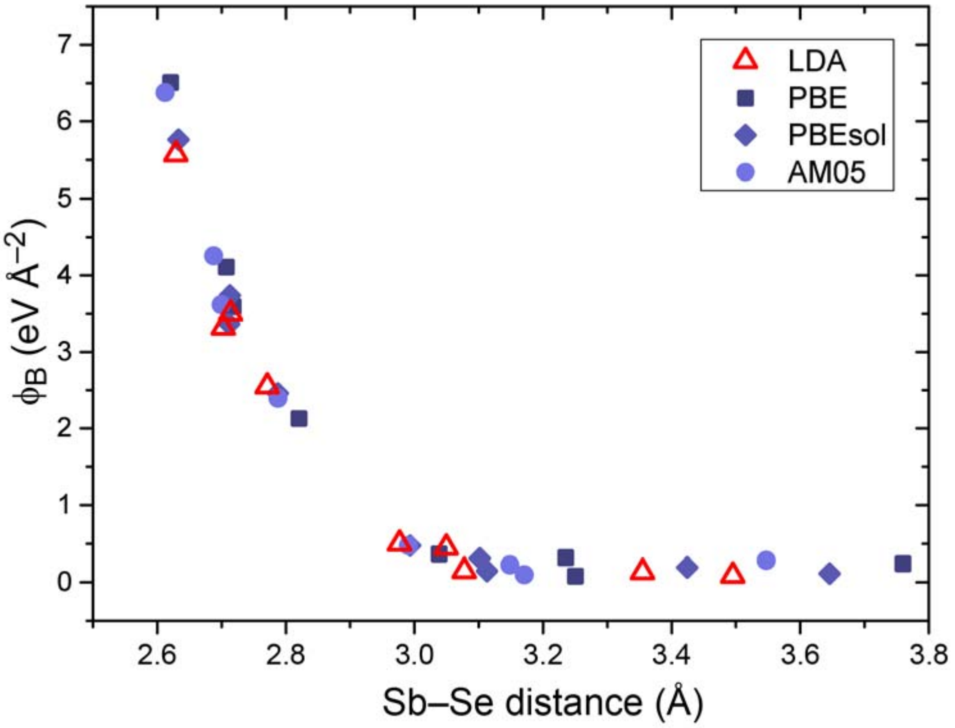

Figure S3 *(as supplement to Figure 7 of the main text)***.** Bond-projected force constants, computed with different GGA functionals. The LDA data used in the main text are given by red triangles for comparison.

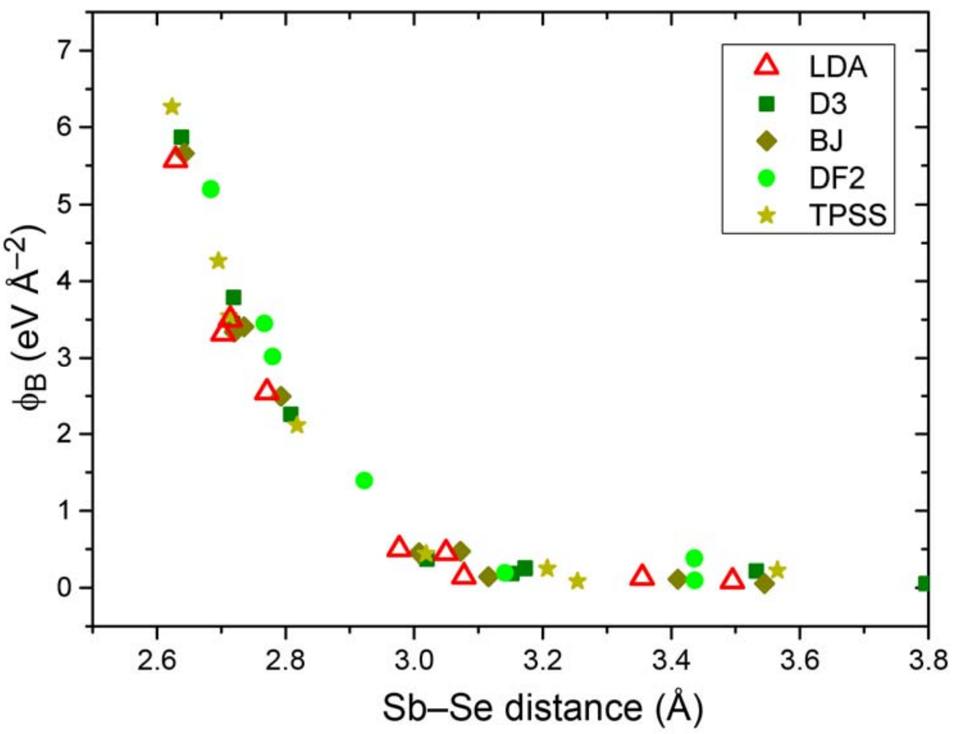

Figure S4 *(as supplement to Figure 7 of the main text)***.** Bond-projected force constants, computed with different dispersion-corrected DFT methods and at the meta-GGA (TPSS) level. The LDA data used in the main text are given by red triangles for comparison.

Supplementary results for bulk Sb_2Se_3

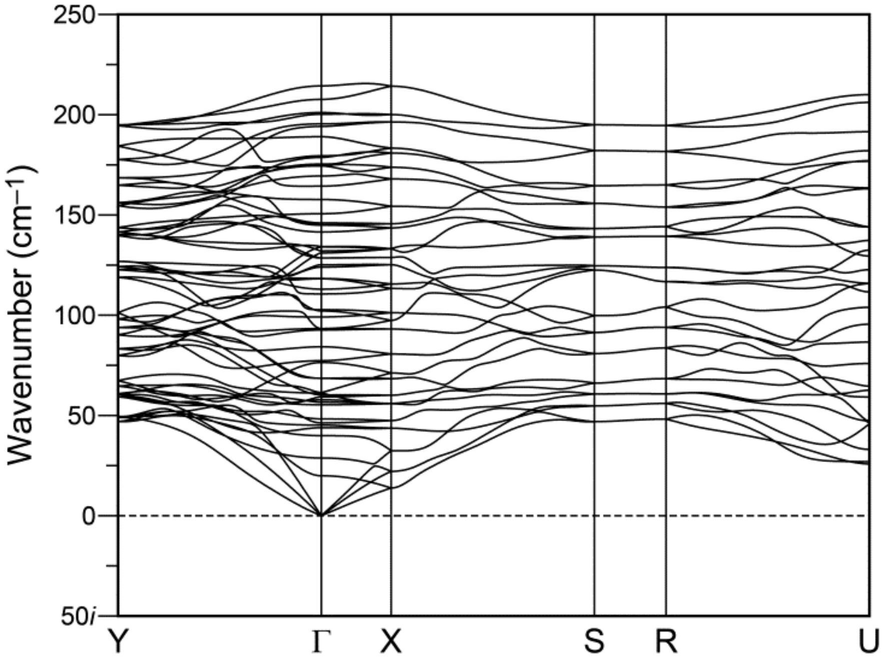

Figure S5 *(as supplement to Figure 5 of the main text)*. Computed phonon band structure along the more comprehensive reciprocal-space pathway that had also been used for the electronic bands in Figure 2. No imaginary contributions are seen over the entire range.

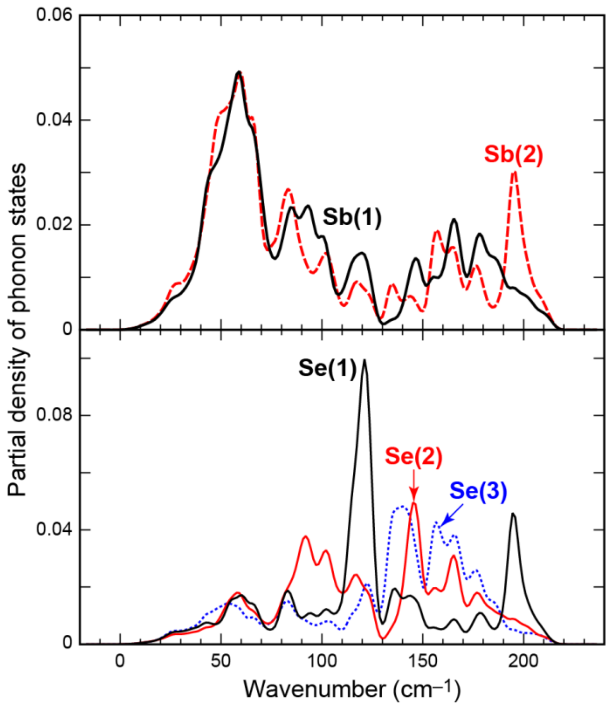

Figure S6 *(as supplement to Figure 5 of the main text)*. Computed partial density of phonon states, resolved according to symmetry-inequivalent antimony (*top*) and selenium (*bottom*) atoms. Note the pronounced contribution of both Sb(2) and Se(1) at around 200 cm^{-1}, corresponding to a stretching vibration of the shortest covalent bond. The large partial density of states of Se(1) at $\approx$ 120 cm^{-1} also seems an interesting target for experimental investigation. The vibrations projected on the antimony atoms (top panel), which lie in the "interior" of the chains, are more evenly distributed over the range of wavenumbers.

Born effective charges. To better understand the bonding nature of the compound, we computed Born effective charges, using density-functional perturbation theory (DFPT)[S15] as implemented in VASP (settings: `LEPSILON = .TRUE., LRPA = .FALSE.`).[S16] The analysis of Born effective charges also in comparison to experiment has recently proven a valuable tool to elucidate the nature of bulk and nanoscale oxides and heavier chalcogenides.[S17] Here, doing so is especially interesting as it allows for comparison with recent results for Sb_2S_3.[S18] The computed Born effective charges are listed in **Table S1**, and throughout, they are slightly larger than those of the lighter sulphide compound.[S18]

Table S1 *(as supplement to Figure 5 of the main text).* Nonzero elements of the Born effective charge tensor as computed here for Sb_2Se_3. The presentation is similar to that in Ref. S18, to ease comparison with the results for Sb_2S_3 given in the latter work.

	xx	*yy*	*zz*	*xz*	*zx*
Sb(1)	3.07	5.92	8.17	0.25	1.87
Sb(2)	2.98	7.45	4.87	0.46	0.20
Se(1)	–2.02	–4.23	–4.71	1.19	1.11
Se(2)	–1.84	–5.09	–4.98	–0.27	–0.13
Se(3)	–2.16	–4.05	–3.37	–0.16	–1.62

Force constants and Badger's relation. Here, we provide raw data for the bond-projected force constants as discussed in the main text, and also supply additional data to further justify the hypothesis of low force constants in the "weak" Sb–Se bonds. According to Badger, the force constants ϕ and bond distances d in molecules obey[S19]

$$\phi \times (d - d_0)^3 = \text{const.}$$

where d_0 is an element-specific constant. Plotting the cubic root of ϕ^{-1} hence yields a linear relationship if Badger's relation is satisfied. Doing so for the dataset obtained here for Sb_2Se_3 reveals that the short bonds within the 1D chains appear to follow a Badger-like relation, whereas the weaker bonds deviate because their force constants are lower (**Figure S7**).

Table S2 *(as supplement to Figure 7 of the main text)*. Bond-projected force constants.

	d_{opt} (Å)	ϕ_B (eV Å^{-2})
Sb(2)–Se(1)	2.629	5.572
Sb(1)–Se(3)	2.714	3.500
Sb(1)–Se(2)	2.703	3.315
Sb(2)–Se(3)	2.771	2.545
Sb(2)–Se(1)'	2.977	0.306
Sb(1)–Se(1)	3.077	0.267
Sb(1)–Se(2)'	3.049	0.444
Sb(2)–Se(2)'	3.355	0.070
Sb(1)–Se(3)'	3.495	0.084

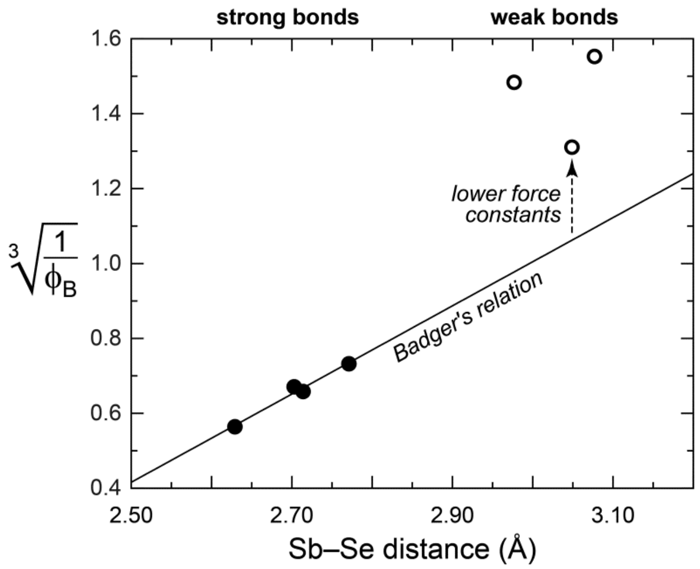

Figure S7 *(as supplement to Figure 7 of the main text)*. Application of Badger's relation to the short and medium-range bonds in Sb_2Se_3.

Supplementary results for 1D chains

Dynamic stability of low-dimensional fragments. To further validate the results for 1D fragments cleaved from the crystal structure (Figure 6 in the main text), we have performed additional computations at the GGA (PBEsol) and dispersion-corrected GGA level (using Grimme's "D3" scheme). The resulting phonon band structures are collected in **Figure S8**. For the single wire characterised in panel (a), all three methods arrive at imaginary vibrational wavenumbers, qualitatively confirming the result of the LDA DPS; for the double wire in panel (b), all three methods evidence dynamic stability. For the chains bridged *via* longer interchain contacts, the PBEsol approach confirms the LDA result, albeit we note that a trace amount of imaginary modes does appear in the dispersion-corrected result.

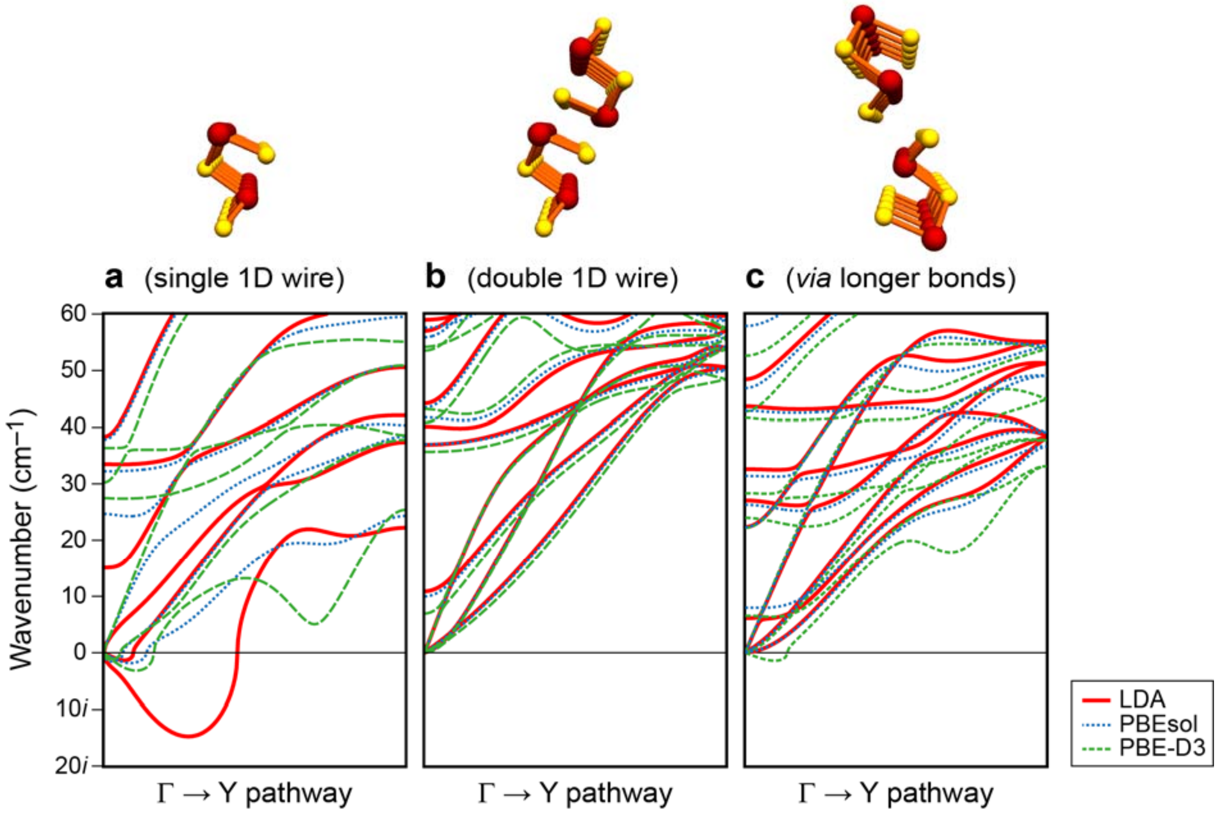

Figure S8 *(as supplement to Figure 6 of the main text)*. Phonon band structure for computations on 1D fragments as described in the main text, showing results for (a) a single wire, (b) a double wire bridged *via* the "primary inter-chain" bond as defined in the main text, and (c) a double wire bridged via the "secondary inter-chain" bond. Results from the LDA (red solid lines), the GGA functional PBEsol (blue dotted lines) and the dispersion-corrected PBE-D3 approach (green dashed lines) are provided.

The results provided so far have been obtained for structural fragments from the optimised crystal structure. However, subsequent optimisation of the 1D models is also an option: this alters the structural details compared to the bulk network, but it can provide additional insight regarding the dynamic stability. Results for these fully re-optimised models are given in **Figure S9**: again, a dynamic instability is observed for the single chain at all three levels of theory.

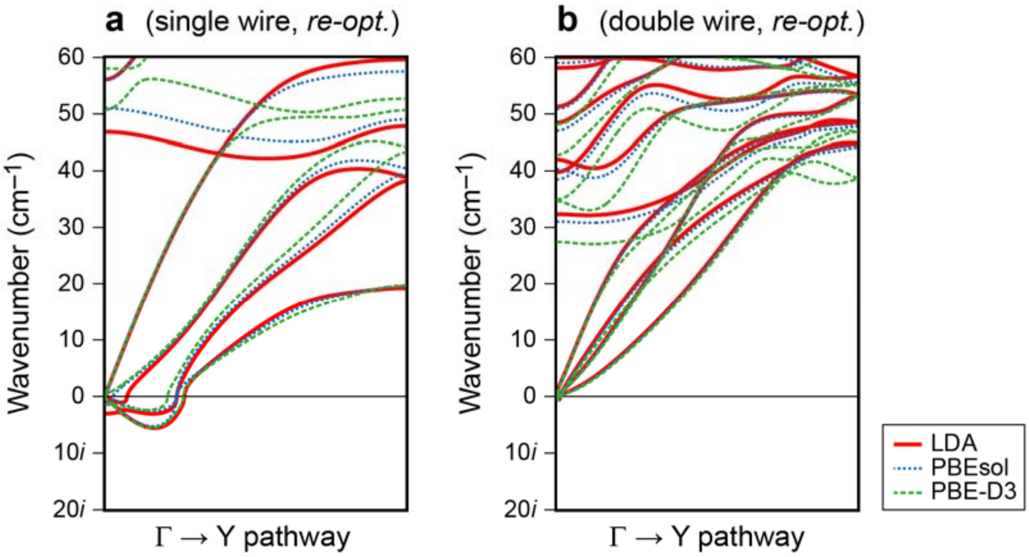

Figure S9 *(as supplement to Figure 6 of the main text)*. Phonon band structures as before, but for fully re-optimised models at the respective level of theory. The double wire bridged via longer bonds relaxed into a distinctly different, but ultimately unstable structure; it is hence not considered here. In the double wire characterised in panel (b), on the contrary, the character of the "interchain" contacts remained similar, changing only from 2.977 Å to 2.960 Å (LDA), for example.

Structural raw data

Listing S1. Optimised structure of Sb_2Se_3 at the LDA-DFT level. Lattice vectors and coordinates are provided in VASP `POSCAR` format for convenience.

```
Antimony selenide LDA
   1.00000000000000
    11.5336666436761384    0.0000000000000000    0.0000000000000000
     0.0000000000000000    3.9601337536816592    0.0000000000000000
     0.0000000000000000    0.0000000000000000   11.2210518223176425
   Sb   Se
     8    12
Direct
  0.1532363560821750  0.7500000000000000  0.9621619931950463
  0.5187766115735997  0.2500000000000000  0.8283942748294990
  0.0187766115735997  0.2500000000000000  0.6716057251705010
  0.6532363560821750  0.7500000000000000  0.5378380068049537
  0.3467636439178250  0.2500000000000000  0.4621619931950463
  0.9812233884264003  0.7500000000000000  0.3283942748294990
  0.4812233884264003  0.7500000000000000  0.1716057251705010
  0.8467636439178250  0.2500000000000000  0.0378380068049537
  0.6294130243619236  0.7500000000000000  0.9465496402764586
  0.9448073833154211  0.7500000000000000  0.8673043928942263
  0.2168040381329206  0.2500000000000000  0.8022423206686895
  0.7168040381329206  0.2500000000000000  0.6977576793313105
  0.4448073833154211  0.7500000000000000  0.6326956071057737
  0.1294130243619236  0.7500000000000000  0.5534503597235414
  0.8705869756380764  0.2500000000000000  0.4465496402764586
  0.5551926166845789  0.2500000000000000  0.3673043928942263
  0.2831959618670794  0.7500000000000000  0.3022423206686966
  0.7831959618670794  0.7500000000000000  0.1977576793313105
  0.0551926166845718  0.2500000000000000  0.1326956071057737
  0.3705869756380764  0.2500000000000000  0.0534503597235414
```

Listing S2. As before, but at the PBE level.

```
Antimony selenide PBE
   1.00000000000000
    12.8489545679032773    0.0000000000000000    0.0000000000000000
     0.0000000000000000    4.0301506162767247    0.0000000000000000
     0.0000000000000000    0.0000000000000000   11.5352420140232397
   Sb   Se
     8    12
Direct
  0.1373604255775902  0.7500000000000000  0.9632718044882012
  0.5356406967537808  0.2500000000000000  0.8319871483283805
  0.0356406967537808  0.2500000000000000  0.6680128516716195
  0.6373604255775902  0.7500000000000000  0.5367281955117988
  0.3626395744224098  0.2500000000000000  0.4632718044881941
  0.9643593032462192  0.7500000000000000  0.3319871483283805
  0.4643593032462192  0.7500000000000000  0.1680128516716195
  0.8626395744224098  0.2500000000000000  0.0367281955117988
  0.6274410940012274  0.7500000000000000  0.9525940357710780
  0.9526080941564530  0.7500000000000000  0.8669704722851250
  0.2040079922379832  0.2500000000000000  0.8091468161273951
  0.7040079922379761  0.2500000000000000  0.6908531838726049
  0.4526080941564530  0.7500000000000000  0.6330295277148750
  0.1274410940012203  0.7500000000000000  0.5474059642289220
  0.8725589059987726  0.2500000000000000  0.4525940357710851
  0.5473919058435470  0.2500000000000000  0.3669704722851250
  0.2959920077620168  0.7500000000000000  0.3091468161273951
  0.7959920077620239  0.7500000000000000  0.1908531838726049
  0.0473919058435470  0.2500000000000000  0.1330295277148750
  0.3725589059987797  0.2500000000000000  0.0474059642289149
```

Listing S3. As before, but at the PBEsol level.

```
Antimony selenide PBEsol
   1.00000000000000
    11.7891100604765793    0.0000000000000000    0.0000000000000000
     0.0000000000000000    3.9797741767087893    0.0000000000000000
     0.0000000000000000    0.0000000000000000   11.3175337334822466
   Sb   Se
     8    12
Direct
  0.1502664476415774  0.7500000000000000  0.9628785179197905
  0.5231093738531527  0.2500000000000000  0.8286296588467152
  0.0231093738531527  0.2500000000000000  0.6713703411532848
  0.6502664476415774  0.7500000000000000  0.5371214820802095
  0.3497335523584226  0.2500000000000000  0.4628785179197905
  0.9768906261468473  0.7500000000000000  0.3286296588467152
  0.4768906261468473  0.7500000000000000  0.1713703411532848
  0.8497335523584226  0.2500000000000000  0.0371214820802095
  0.6293574757508864  0.7500000000000000  0.9480210106325728
  0.9466475465424224  0.7500000000000000  0.8672883123855755
  0.2147524380337984  0.2500000000000000  0.8040554669344786
  0.7147524380337984  0.2500000000000000  0.6959445330655214
  0.4466475465424224  0.7500000000000000  0.6327116876144245
  0.1293574757508864  0.7500000000000000  0.5519789893674272
  0.8706425242491136  0.2500000000000000  0.4480210106325728
  0.5533524534575776  0.2500000000000000  0.3672883123855826
  0.2852475619662087  0.7500000000000000  0.3040554669344786
  0.7852475619662016  0.7500000000000000  0.1959445330655214
  0.0533524534575776  0.2500000000000000  0.1327116876144174
  0.3706425242491136  0.2500000000000000  0.0519789893674272
```

Listing S4. As before, but at the AM05 level.

```
Antimony selenide AM05
   1.00000000000000
    12.2211556879451777    0.0000000000000000    0.0000000000000000
     0.0000000000000000    3.9767652246593932    0.0000000000000000
     0.0000000000000000    0.0000000000000000   11.3647823107411163
   Sb   Se
     8    12
Direct
  0.1442912063034143  0.7500000000000000  0.9643954643518597
  0.5301888777297421  0.2500000000000000  0.8294998967016056
  0.0301888777297421  0.2500000000000000  0.6705001032983944
  0.6442912063034143  0.7500000000000000  0.5356045356481403
  0.3557087936965857  0.2500000000000000  0.4643954643518597
  0.9698111222702579  0.7500000000000000  0.3294998967016056
  0.4698111222702579  0.7500000000000000  0.1705001032983944
  0.8557087936965857  0.2500000000000000  0.0356045356481403
  0.6288892605501673  0.7500000000000000  0.9500870167622892
  0.9504734426217496  0.7500000000000000  0.8674395241861532
  0.2095061043178958  0.2500000000000000  0.8074203731107445
  0.7095061043178958  0.2500000000000000  0.6925796268892555
  0.4504734426217496  0.7500000000000000  0.6325604758138468
  0.1288892605501815  0.7500000000000000  0.5499129832377108
  0.8711107394498327  0.2500000000000000  0.4500870167622821
  0.5495265573782504  0.2500000000000000  0.3674395241861532
  0.2904938956821042  0.7500000000000000  0.3074203731107445
  0.7904938956821042  0.7500000000000000  0.1925796268892555
  0.0495265573782504  0.2500000000000000  0.1325604758138468
  0.3711107394498185  0.2500000000000000  0.0499129832377179
```

Listing S5. As before, but at the PBE-D3 (zero-damping) level.

```
Antimony selenide PBE-D3
   1.00000000000000
    12.0470523636550180    0.0000000000000000    0.0000000000000000
     0.0000000000000000    4.0188925464843619    0.0000000000000000
     0.0000000000000000    0.0000000000000000   11.4593148812205037
   Sb   Se
     8    12
Direct
  0.1465231278037393  0.7500000000000000  0.9618922440694675
  0.5267211888437373  0.2500000000000000  0.8295055370489237
  0.0267211888437373  0.2500000000000000  0.6704944629510763
  0.6465231278037322  0.7500000000000000  0.5381077559305325
  0.3534768721962607  0.2500000000000000  0.4618922440694675
  0.9732788111562627  0.7500000000000000  0.3295055370489237
  0.4732788111562627  0.7500000000000000  0.1704944629510763
  0.8534768721962678  0.2500000000000000  0.0381077559305325
  0.6278611326473964  0.7500000000000000  0.9485748683935498
  0.9467587725350484  0.7500000000000000  0.8675639325439377
  0.2124412174909409  0.2500000000000000  0.8053437669114913
  0.7124412174909409  0.2500000000000000  0.6946562330885087
  0.4467587725350413  0.7500000000000000  0.6324360674560623
  0.1278611326473964  0.7500000000000000  0.5514251316064502
  0.8721388673526036  0.2500000000000000  0.4485748683935498
  0.5532412274649516  0.2500000000000000  0.3675639325439377
  0.2875587825090591  0.7500000000000000  0.3053437669114913
  0.7875587825090591  0.7500000000000000  0.1946562330885087
  0.0532412274649516  0.2500000000000000  0.1324360674560623
  0.3721388673526036  0.2500000000000000  0.0514251316064502
```

Listing S6. As before, but at the PBE-D3 level using Becke–Johnson damping.

```
Antimony selenide PBE-D3(BJ)
   1.00000000000000
    11.6609613152934326    0.0000000000000000    0.0000000000000000
     0.0000000000000000    3.9894602340440857    0.0000000000000000
     0.0000000000000000    0.0000000000000000   11.3468039010953223
   Sb   Se
     8    12
Direct
  0.1518177384332944  0.7500000000000000  0.9605701407281018
  0.5190404680656400  0.2500000000000000  0.8291023668929896
  0.0190404680656400  0.2500000000000000  0.6708976331070104
  0.6518177384332944  0.7500000000000000  0.5394298592718982
  0.3481822615667056  0.2500000000000000  0.4605701407281018
  0.9809595319343600  0.7500000000000000  0.3291023668929896
  0.4809595319343600  0.7500000000000000  0.1708976331070104
  0.8481822615667056  0.2500000000000000  0.0394298592718982
  0.6287897735184060  0.7500000000000000  0.9467996737548745
  0.9441757687545973  0.7500000000000000  0.8672849872254176
  0.2162266264795818  0.2500000000000000  0.8014884017968456
  0.7162266264795818  0.2500000000000000  0.6985115982031544
  0.4441757687545973  0.7500000000000000  0.6327150127745824
  0.1287897735184060  0.7500000000000000  0.5532003262451255
  0.8712102264815940  0.2500000000000000  0.4467996737548745
  0.5558242312454027  0.2500000000000000  0.3672849872254176
  0.2837733735204182  0.7500000000000000  0.3014884017968456
  0.7837733735204182  0.7500000000000000  0.1985115982031544
  0.0558242312454027  0.2500000000000000  0.1327150127745824
  0.3712102264815940  0.2500000000000000  0.0532003262451255
```

Listing S7. As before, but at the vdW-DF2 level.

```
Antimony selenide vdW-DF2
   1.00000000000000
    12.8607238342795203    0.0000000000000000    0.0000000000000000
     0.0000000000000000    4.1797315381310387    0.0000000000000000
     0.0000000000000000    0.0000000000000000   12.1770930122101824
   Sb   Se
     8    12
Direct
  0.1377869708752471  0.7500000000000000  0.9590881212288949
  0.5387336950931427  0.2500000000000000  0.8315580085590213
  0.0387336950931427  0.2500000000000000  0.6684419914409787
  0.6377869708752471  0.7500000000000000  0.5409118787711051
  0.3622130291247601  0.2500000000000000  0.4590881212288949
  0.9612663049068573  0.7500000000000000  0.3315580085590213
  0.4612663049068573  0.7500000000000000  0.1684419914409787
  0.8622130291247529  0.2500000000000000  0.0409118787711051
  0.6286120421170480  0.7500000000000000  0.9484307444578022
  0.9467965129682199  0.7500000000000000  0.8703037768068285
  0.2080969777661537  0.2500000000000000  0.8086130044735569
  0.7080969777661537  0.2500000000000000  0.6913869955264431
  0.4467965129682199  0.7500000000000000  0.6296962231931715
  0.1286120421170409  0.7500000000000000  0.5515692555421978
  0.8713879578829520  0.2500000000000000  0.4484307444578022
  0.5532034870317801  0.2500000000000000  0.3703037768068285
  0.2919030222338463  0.7500000000000000  0.3086130044735640
  0.7919030222338463  0.7500000000000000  0.1913869955264360
  0.0532034870317801  0.2500000000000000  0.1296962231931715
  0.3713879578829591  0.2500000000000000  0.0515692555421978
```

Listing S8. As before, but at the TPSS level.

```
Antimony selenide TPSS
   1.00000000000000
    12.2349286380501052    0.0000000000000000    0.0000000000000000
     0.0000000000000000    4.0124881993604848    0.0000000000000000
     0.0000000000000000    0.0000000000000000   11.5950078194070993
   Sb   Se
     8    12
Direct
  0.1453269833984692  0.7500000000000000  0.9639480175228883
  0.5312660554942497  0.2500000000000000  0.8284474713919678
  0.0312660554942497  0.2500000000000000  0.6715525286080322
  0.6453269833984692  0.7500000000000000  0.5360519824771117
  0.3546730166015379  0.2500000000000000  0.4639480175228883
  0.9687339445057503  0.7500000000000000  0.3284474713919678
  0.4687339445057503  0.7500000000000000  0.1715525286080322
  0.8546730166015308  0.2500000000000000  0.0360519824771117
  0.6288965432864515  0.7500000000000000  0.9475077438357431
  0.9503427503673905  0.7500000000000000  0.8697650177755847
  0.2100143911952301  0.2500000000000000  0.8074889714779587
  0.7100143911952230  0.2500000000000000  0.6925110285220413
  0.4503427503673905  0.7500000000000000  0.6302349822244153
  0.1288965432864515  0.7500000000000000  0.5524922561642569
  0.8711034567135485  0.2500000000000000  0.4475077438357431
  0.5496572496326095  0.2500000000000000  0.3697650177755847
  0.2899856088047699  0.7500000000000000  0.3074889714779587
  0.7899856088047770  0.7500000000000000  0.1925110285220413
  0.0496572496326095  0.2500000000000000  0.1302349822244153
  0.3711034567135485  0.2500000000000000  0.0524922561642569
```

Supplementary references

(S1) R. P. Stoffel, V. L. Deringer, R. E. Simon, R. P. Hermann and R. Dronskowski, *J. Phys.: Condens. Matter*, 2015, **27**, 085402.

(S2) J. P. Perdew and K. Schmidt, ed. V. Van Doren et al., American Institute of Physics, Editon edn., 2001, pp. 1-20.

(S3) J. P. Perdew, K. Burke and M. Ernzerhof, *Phys. Rev. Lett.*, 1996, **77**, 3865-3868.

(S4) J. P. Perdew, A. Ruzsinszky, G. I. Csonka, O. A. Vydrov, G. E. Scuseria, L. A. Constantin, X. Zhou and K. Burke, *Phys. Rev. Lett.*, 2008, **100**, 136406.

(S5) R. Armiento and A. E. Mattsson, *Phys. Rev. B*, 2005, **72**, 085108.

(S6) M. R. Filip, C. E. Patrick and F. Giustino, *Phys. Rev. B*, 2013, **87**, 205125.

(S7) S. Grimme, J. Antony, S. Ehrlich and H. Krieg, *J. Chem. Phys.*, 2010, **132**, 154104.

(S8) S. Grimme, S. Ehrlich and L. Goerigk, *J. Comput. Chem.*, 2011, **32**, 1456-1465.

(S9) (a) M. Dion, H. Rydberg, E. Schröder, D. C. Langreth and B. I. Lundqvist, *Phys. Rev. Lett.*, 2004, **92**, 246401; (b) K. Lee, É. D. Murray, L. Kong, B. I. Lundqvist and D. C. Langreth, *Phys. Rev. B*, 2010, **82**, 081101(R); (c) J. Klimeš, D. R. Bowler and A. Michaelides, *Phys. Rev. B*, 2011, **83**, 195131.

(S10) J. Tao, J. P. Perdew, V. N. Staroverov and G. E. Scuseria, *Phys. Rev. Lett.*, 2003, **91**, 146401.

(S11) T. Björkman, A. Gulans, A. V. Krasheninnikov and R. M. Nieminen, *J. Phys.: Condens. Matter*, 2012, **24**, 424218.

(S12) G. P. Voutsas, A. G. Papazoglou, P. J. Rentzeperis and D. Siapkas, *Z. Kristallogr.*, 1985, **171**, 261-268.

(S13) J. van de Streek and M. A. Neumann, *Acta Crystallogr., Sect. B*, 2010, **66**, 544-558.

(S14) J. George, V. L. Deringer and R. Dronskowski, *Inorg. Chem.*, 2015, **54**, 956-962.

(S15) S. Baroni, S. de Gironcoli, A. Dal Corso and P. Giannozzi, *Rev. Mod. Phys.*, 2001, **73**, 515-562.

(S16) M. Gajdoš, K. Hummer, G. Kresse, J. Furthmüller and F. Bechstedt, *Phys. Rev. B*, 2006, **73**, 045112.

(S17) (a) Q.-C. Sun, X. S. Xu, L. I. Vergara, R. Rosentsveig and J. L. Musfeldt, *Phys. Rev. B*, 2009, **79**, 205405; (b) Q.-C. Sun, X. Xu, S. N. Baker, A. D. Christianson and J. L. Musfeldt, *Chem. Mater.*, 2011, **23**, 2956-2960.

(S18) Y. Liu, K. T. E. Chua, T. C. Sum and C. K. Gan, *Phys. Chem. Chem. Phys.*, 2014, **16**, 345-350.

(S19) R. M. Badger, *J. Chem. Phys.*, 1934, **2**, 128-131.
